# Seasonal and Spatial Variations in *Synechococcus* Abundance and Diversity Throughout the Gullmar Fjord, Swedish Skagerrak

**DOI:** 10.3389/fmicb.2022.828459

**Published:** 2022-05-09

**Authors:** Christien P. Laber, Benjamin Pontiller, Carina Bunse, Christofer M. G. Osbeck, Clara Pérez-Martínez, Danilo Di Leo, Daniel Lundin, Catherine Legrand, Jarone Pinhassi, Hanna Farnelid

**Affiliations:** ^1^Centre for Ecology and Evolution in Microbial Model Systems – EEMiS, Department of Biology and Environmental Science, Linnaeus University, Kalmar, Sweden; ^2^GEOMAR Helmholtz Centre for Ocean Research Kiel, Kiel, Germany; ^3^Helmholtz Institute for Functional Marine Biodiversity at the University of Oldenburg (HIFMB), Oldenburg, Germany; ^4^Institute for Chemistry and Biology of the Marine Environment (ICBM), University of Oldenburg, Oldenburg, Germany; ^5^School of Business, Innovation and Sustainability, Halmstad University, Halmstad, Sweden

**Keywords:** *Synechococcus*, Gullmar Fjord, microbial ecology, seasonal succession, picophytoplankton, ecotype

## Abstract

The picophytoplankton *Synechococcus* is a globally abundant autotroph that contributes significantly to primary production in the oceans and coastal areas. These cyanobacteria constitute a diverse genus of organisms that have developed independent niche spaces throughout aquatic environments. Here, we use the 16S V3–V4 rRNA gene region and flow cytometry to explore the diversity of *Synechococcus* within the picophytoplankton community in the Gullmar Fjord, on the west coast of Sweden. We conducted a station-based 1-year time series and two transect studies of the fjord. Our analysis revealed that within the large number of *Synechococcus* amplicon sequence variants (ASVs; 239 in total), prevalent ASVs phylogenetically clustered with clade representatives in both marine subcluster 5.1 and 5.2. The near-surface composition of ASVs shifted from spring to summer, when a 5.1 subcluster dominated community developed along with elevated *Synechococcus* abundances up to 9.3 × 10^4^ cells ml^–1^. This seasonal dominance by subcluster 5.1 was observed over the length of the fjord (25 km), where shifts in community composition were associated with increasing depth. Unexpectedly, the community shift was not associated with changes in salinity. *Synechococcus* abundance dynamics also differed from that of the photosynthetic picoeukaryote community. These results highlight how seasonal variations in environmental conditions influence the dynamics of *Synechococcus* clades in a high latitude threshold fjord.

## Introduction

Picophytoplankton are globally important primary producers with cell sizes <2 μm in diameter and are estimated to make up a third or more of phytoplankton biomass in the world’s oceans (Quere et al., 2005; Buitenhuis et al., 2012). Cyanobacteria in this category of phytoplankton, typically referred to as picocyanobacteria, include the genera *Synechococcus*, *Prochlorococcus*, and *Cyanobium*. While *Prochlorococcus* distribution is limited to warmer low-mid latitude open ocean environments (Johnson et al., 2006), *Synechococcus* exhibits a geographic distribution that extends to polar latitudes and high nutrient coastal systems (Partensky et al., 1999). Further, *Synechococcus* is a dominant picophytoplankton in freshwater systems, often sharing the environment with *Cyanobium* (Callieri, 2008). With this broad realized niche space, it is estimated that *Synechococcus* is responsible for 17% of global ocean net primary production (Flombaum et al., 2013).

The success of *Synechococcus* in diverse ecosystems ranging from the nutrient poor open ocean to freshwater lakes is attributed to extensive niche partitioning that has been observed within the genus (Ferris and Palenik, 1998). Physiological expression of these adaptations includes chromatic adaptations through modulation of accessory pigments (Palenik, 2001), motility (Waterbury et al., 1985), and specialized nutrient acquisition (Moore et al., 2002). The 16S rRNA gene was initially useful in classifying marine *Synechococcus* into three subclusters numbered 5.1, 5.2, and 5.3, and the resolution of this diversity has been further resolved using additional marker genes including 16S-23S internally transcribed spacer (ITS) regions, and the *narB*, *rpoC1*, and *ntcA* genes (Ahlgren and Rocap, 2012). These additional markers have resolved at least 20 distinct clades within the three subclusters and have been used to identify trends in environmental partitioning among the clades (Zwirglmaier et al., 2007; Tai et al., 2011). While geographical distributions are flexible, the subcluster 5.1 clades I and IV have been identified as being prominent in colder, high latitude waters, while clade III dominates in tropical and subtropical oligotrophic waters (Zwirglmaier et al., 2008; Mazard et al., 2012). Notably, clades I and IV also provide an example of subcluster 5.1A and 5.1B lineage co-occurrence, where separate lineages come to concurrently occupy similar niche space via independent evolutionary pathways (Zwirglmaier et al., 2008; Mazard et al., 2012). Subcluster 5.2 also exhibits geographic partitioning, with member clades largely found in temperate coastal waters (Huang et al., 2012). Notably, marine subcluster 5.2 also forms a sister cluster with some lake and low salinity brackish water strains (Callieri et al., 2013). While niche partitioning and co-occurrences are commonly observed, there is still limited understanding of the factors that control clade abundances and distribution.

Estuarine systems often have distinct and heterogeneous *Synechococcus* community structure in comparison to marine environments. This is due to the mixed influence from riverine and offshore water sources creating environmental fluctuations and gradients within the system. Larger systems such as the Chesapeake Bay and Pearl River Estuary exhibit changes in community composition from coastal to upper estuary sites (Chen et al., 2006; Xia et al., 2017). These studies found fresh water and marine subcluster 5.2 *Synechococcus* dominant in the upper estuaries while subcluster 5.1 was dominant in the coastal environment. This difference in community structure has been linked to salinity in the Pearl River system (Xia et al., 2017), and indeed, this transition from subcluster 5.2 to 5.1 is even observed in the larger horizontal salinity gradient of the Baltic Sea (Celepli et al., 2017). However, in smaller estuaries such as Little Sippewissett salt marsh, dynamics in the main channel more closely reflected the marine source water and changes in community structure were more closely related to seasonal temperature and salinity (Mackey et al., 2017).

Future climate projections suggest increased relative and overall contribution of picophytoplankton to ocean primary production (Morán et al., 2010; Flombaum et al., 2020; Visintini et al., 2021) with *Synechococcus* distributions possibly further extending at high latitudes (Flombaum et al., 2013) or increasing in abundances where they are already prevalent (Flombaum et al., 2020). This reveals a need to further understand the geographic and temporal dynamics of this genus. Moreover, data informing these models are mostly derived from lower latitudes (Visintini et al., 2021) and therefore more high latitude marine observations are necessary to maximize their predictive value.

This study investigated the contribution of *Synechococcus* to the picoplankton microbial community in the Gullmar Fjord, Sweden, in a yearlong time series and transects taking place in 2016–2017. Specifically, we explored seasonal abundance of picophytoplankton, discriminating *Synechococcus* from photosynthetic picoeukaryotes (PPE)s, and the spatial niche partitioning of *Synechococcus* subclusters and clades within the fjord’s dynamic salinity and temperature environment. This threshold fjord (25 km long, 1–3 km wide, 45–125 m deep) is located on the west coast of Sweden at the junction of the Kattegat and Skagerrak and is influenced by lower salinity waters coming from the Baltic Sea and fully marine salinity waters from the Skagerrak as well as river water from the Örekilsälven (Svansson, 1984). It is a well-studied fjord with regular monitoring of primary production near the inlet that dates back to 1985 (Lindahl et al., 2009). We examined the *Synechococcus* community structure using MiSeq Illumina high throughput sequencing of the V3–V4 region of 16S rRNA gene. These data provide an important contribution to studying the diversity and distribution of *Synechococcus*, particularly in high-latitude marine coastal systems.

## Materials and Methods

### Sampling

Surface water was collected from the Pricken station in the Gullmar Fjord (58°15.4′N, 11°27.1′E) between the dates of January 18, 2016, to March 21, 2017, with sampling every 2–4 weeks throughout the time series. Seawater was collected at 2–10 m from the surface at 2 m intervals. This water was then pooled to represent the average surface water for heterotrophic and phototrophic bacterial DNA and flow cytometry counts. Samples for bacterial DNA were collected by filtering 0.5–2 L seawater through 0.2 μm pore-size 47 mm Supor^(R)^-200 membrane filters (Pall, Life Sciences, United States), filters were subsequently stored in 1.8 ml 1 × TE-buffer (sterile-filtered) and frozen at −80°C until further processing. All conductivity, temperature, and density (CTD) measurements and Chlorophyll *a* (Chl *a*) fluorescence measurements from the time series profiles were averaged from 2 to 10 m, corresponding to the integrated depth range.

**FIGURE 1:**
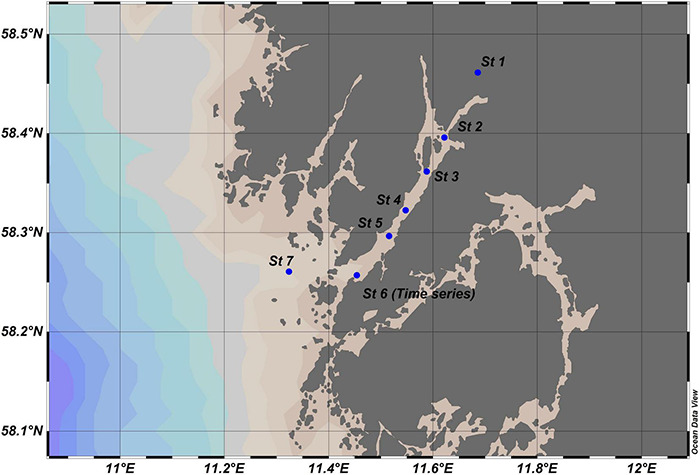
Map of the sampling locations in the Gullmar Fjord (St. 1–St. 7). The 13-month time series in 2016–2017 occurred in the mouth of the fjord (St. 6). The two transects that sampled the multiple depths over the water column occurred in July (KB1707) and September (KB1709) in 2017 at seven stations along the fjord with St. 1 positioned in the Örekilsälven river and St. 7 positioned at the entry to the Skagerrak Strait.

The two transect studies of the Gullmar Fjord took place on July 13 and September 18, 2017, with R/V Oscar von Sydow (hereafter referred to as Summer KB1707 and Fall KB1709 transects). These transects were composed of seven stations distributed from the Örekilsälven river (2 km up river) to a station located 5 km outside the mouth of the fjord. The river station was sampled just below surface by filling 10 L carboys, and samples were subsequently processed in the laboratory. Whole water column sampling of the remaining stations was conducted using Niskin water sampling bottles following CTD casts. We sampled depth profiles at the surface (2–5 m), deep chlorophyll max (12–15 m), and approximately every 15–35 m below (50–100 m) depending on the respective bottom profile, resulting in 3–5 depths per station. Station 6 was the location of the time series sampled in 2016 (Figure 1). Bacterial DNA was sampled at each depth in the same way as for the time series. CTD data from the KB1707 and KB1709 cruise transects were averaged in 1 m depth bins, each including >5 measurements, with profiles sampling the full depth of the water column.

To measure water column nutrients in the transects, approximately ∼500 ml of seawater was collected and stored in PET bottles that were previously washed with 5% HCl and pre-rinsed with Milli-Q and seawater. Nutrient samples were stored at −20°C until further analysis. Aliquots of 12 ml of samples for NO_3_^–^ and NO_2_^–^, NH_4_^+^, PO_4_^3–^, and SiO_2_ were transferred into 13 ml polystyrene tubes (Sarstedt). All nutrients were measured in a QuAATtro AutoAnalyzer and XY−3 Sampler (Seal Analytics, United States) at the Sven Lovén Centre for Marine Infrastructure in Kristineberg, Sweden. Nutrient data from the time series was downloaded from the SHARKweb database^1^, station name Släggö.

To measure Chl *a*, for the transect dataset, 150–300 ml seawater was collected on A/E glass fiber filters. The filters were stored in 5 ml 96% EtOH for 20 h at room temperature according to Jespersen and Christoffersen (1987). Extracted Chl *a* was measured on a Trilogy^(R)^ fluorometer (Turner Designs, United States). Time series measurements of Chl *a* were done according to Tiselius and Møller (2017).

Samples for measuring heterotrophic and phototrophic cell abundances using flow cytometry were collected in 2 ml cryovials. Samples for bacterial counts were fixed using 1% paraformaldehyde + 0.05% glutaraldehyde final concentration and incubated at room temperature for 10 min. Samples for picophytoplankton (PPEs and *Synechococcus*) counts were fixed with EM-grade glutaraldehyde (final concentration 1%) and incubated at room temperature for 5 min before flash freezing in liquid nitrogen. During the time series, samples were not flash frozen in liquid nitrogen, but directly placed at −80°C. All samples were stored at −80°C until analysis.

### Flow Cytometry

Cells were counted using a Cube8 flow cytometer (Sysmex Partec, Goerlitz, DE, United States). Samples for bacterial abundance were stained with SYBR green (Life Technologies, United States). All samples were collected as two technical replicates. Samples for picophytoplankton abundances were measured unstained using natural pigment fluorescence signals. PPE and *Synechococcus* cells were identified using the forward scatter as a proxy for cell size and the red fluorescence signal from the 488 nm laser as a proxy for Chl *a* content. The two phytoplankton groups were discriminated using FL2 orange fluorescence as a proxy for phycoerythrin pigment present in the *Synechococcus* cells. Gating of cells and abundance measurements were performed in FCSalyzer 0.9.18^2^.

### DNA Extraction

DNA was extracted using a phenol chloroform protocol as described by Boström et al. (2004). Briefly, filters were incubated in TE-buffer and lysozyme (1 mg ml^–1^ final concentration) at 37°C for 30 min. Then, SDS (1% final concentration) and proteinase K (12 mg ml^–1^ final concentration) were added and the filters were incubated overnight at 55°C. The extracts were then transferred to a new tube and equal volumes of phenol chloroform isoamyl alcohol (25:24:1) were added. The samples were centrifuged at 20,000 *g* for 5 min, and the upper phase was collected and placed in a new tube. Equal volumes of chloroform isoamyl alcohol (24:1) were then added to the samples. After a centrifugation at 20,000 *g* for 5 min. the supernatants were collected. DNA was precipitated with 1/10 volume of 3M sodium acetate and 0.6 volume of cold isopropanol at −20°C for an hour and then centrifuged at 4°C, 20,000 *g* for 20 min. The liquid phase was removed, and 500 μl of 70% cold ethanol was added. The samples were centrifuged at 4°C, 20,000 *g* for 20 min, and the liquid removed. The pellet was then dried, and the DNA was re-suspended in 30 μl of TE buffer and stored at −20°C until further processing.

For bacterial community composition analyses, fragments of the 16S rRNA gene were amplified in a two-step PCR procedure followed by high throughput sequencing. In the first round of PCR the 341F and 805R primers were used (Herlemann et al., 2011). Sample specific indexes and adaptors for Illumina sequencing were added in a second PCR. The PCR reactions were prepared in duplicate 25 μl final volume containing 0.25 μM of each primer, 12.5 μl of Phusion Master Mix (Thermo Fisher, United States), 9 μl of pure water and 1 μl of extracted DNA. The PCRs were run with an initial denaturation step at 98°C for 30 s, followed by 20 cycles of the three following steps: 10 s at 98°C, 30 s at annealing temperatures 58°C, and 15 s at 72°C, and finally 2 min at 72°C. PCR amplicons were purified using the Agencourt AMPure XP PCR purification kit. The second PCR reactions were prepared in 25 μl final volume, with 0.25 μM of each primer (adaptors for Illumina sequencing), 12.5 μl of Phusion Master Mix (Thermo Fisher, United States), and 11.5 μl of amplicons from the first PCR. The PCRs were run with an initial denaturation step at 98°C for 30 s, followed by 12 cycles of the three following steps: 10 s at 98°C, 30 s at 62°C, and 5 s at 72°C, and finally 2 min at 72°C. The final PCR products were purified using the Qiagen gel purification kit according to the manufacturer’s instructions to remove residual primers. The concentration and quality of PCR products were measured using NanoDrop and Qubit. Samples were pooled at equal concentrations and were then sequenced using MiSeq Illumina technology (2 × 300 bp) at the National Genomics Infrastructure in Stockholm.

### Sequence and Data Analysis

Raw sequence data was denoised using the ampliseq pipeline v1.2 (Straub et al., 2020) that uses the DADA2 (Callahan et al., 2016) 1.18.0 library implemented in QIIME2 2019.10^3^ (Bolyen et al., 2019). The timeseries library included 2,337,152 reads while the transects library contained 5,627,312 reads. The total number of reads sequenced per sample was between 409,108, and 187 (Avg 65,906 reads). Initial taxonomic assignments were performed with the q2-feature-classifier plugin (Bokulich et al., 2018). The classifier was trained on Silva v132 sequences, trimmed to match the sequence primers. Prior to analyses, sequences classified as of chloroplast and mitochondrial origin were filtered from the dataset. Sequence abundances with taxonomic assignment to Cyanobacteria and Synechococcales were used as filters to compare sequence relative abundances to total bacteria.

To explore *Synechococcus* diversity, amplicon sequence variants (ASVs) were first filtered to retain only those classified in the order Synechococcales. Incomplete sequences were removed from the dataset by filtering out ASVs <390 nt. This resulted in the removal of 2 rare ASVs from the transect datasets and 1 ASV from the time series dataset. An additional 17 ASVs were removed from the dataset after being identified as organisms other than *Synechococcus* by most similar BLAST result (Johnson et al., 2008). For relative abundance measurements, data was exported from qiime2 for processing in RStudio. Duplicate samples were collected for transect samples, and all calculated values represented an average of the two samples (Supplementary Table 1). For each of the datasets (Time series, KB1707, and KB1709), the 20 most represented ASVs were used to explore relative abundances, assisting in visual representation of the datasets. This included all ASVs that made up at least 5% of the Synechococcales ASVs in any individual sample.

### Phylogenetic Trees

To make a phylogenetic tree and assign *Synechococcus* subclusters, a phylogeny was first constructed using 72 full length (∼1,480 bp) 16S rRNA gene *Synechococcus* sp. reference sequences from clades in subclusters 5.1, 5.2, and 5.3. The 16S rRNA gene reference sequences were attained from the NCBI gene database while subcluster and clades were assigned according to Ahlgren and Rocap (2012), Mazard et al. (2012), Choi et al. (2013), and Cabello-Yeves et al. (2017) as well as the Cyanorak database^4^ (Garczarek et al., 2021). The phylogenetic placement tree was obtained by using an in-house Phyton (v.3.9) workflow. A first multiple sequence alignment was obtained with Muscle (v3.8) using the reference sequence (maximum iteration: 2). The alignment was trimmed with TrimAl (v1.4) to obtain a better quality of the final reference tree, as described in Capella-Gutiérrez et al. (2009), with a minimum overlap of positions = 0.55 and a minimum percentage of “good positions” = 60. A maximum-likelihood tree was inferred using the edge-linked partition model in IqTree (Nguyen et al., 2014) with 1,000 bootstraps obtained with ultrafast bootstrap support (Hoang et al., 2017). The parameter model Blosum62 + F + I + G4 was obtained by using the built-in function Modelfinder (Kalyaanamoorthy et al., 2017). To place the ASVs in the reference phylogenetic tree, an alignment between the query fasta sequences and the references was obtained with Hmmalign (HMMER v3.1.b2^5^). EPA-ng (v0.3.8) was then performed with the same model as used in IqTree (Blosum62 + F + I + G4), as suggested in Barbera et al. (2018). ASVs aligned with the same score to multiple reference sequences were placed between the references within the tree. Finally, to visualize the phylogenetic placement, Gappa [v 0.7.1, (Czech et al., 2020)] was used to analyze and convert the jplace file into a text newick file. The phylogenetic placement tree was visualized using the Interactive Tree of Life^6^ (Letunic and Bork, 2019). ASV subclusters were assigned using the known subcluster of the nearest reference strain by branch length. In subcluster 5.1, further classification was possible into 5.1A and 5.1B.

### Statistics

Non-metric multidimensional scaling was performed separately on the time series and transects sequence data using the metaMDS function from the vegan package 2.5−7 in R (Dixon, 2003). The analysis was performed using Bray–Curtis distance and two dimensions of ordination space. The vegan function envfit was used to find the significant correlation of the environmental parameters to the sample ordination.

## Results

### Seawater Temperature, Salinity, and Nutrient Concentrations

**FIGURE 2:**
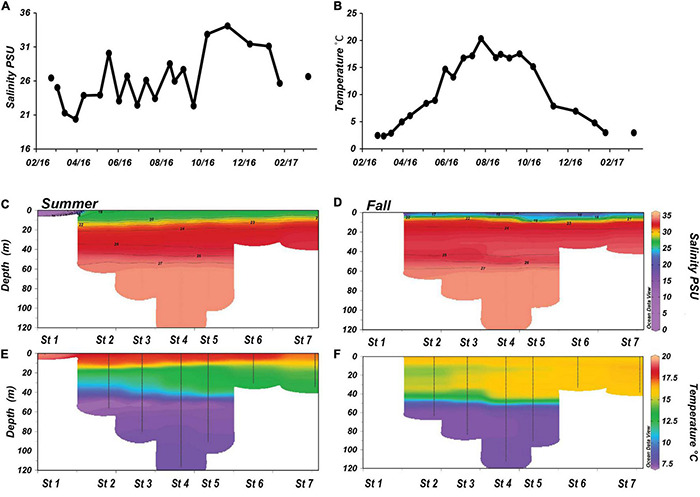
Physical characteristics of Gullmar Fjord during 2016–2017 time series sampling and the summer (KB1707) and fall (KB1709) transect samplings. **(A)** Mean temperature °C and **(B)** salinity measurements of the water column between 2 and 10 m over the duration of the time series sampling. **(C,D)** Salinity measurements of the whole water column during the transects. Superimposed over the salinities are contours of *in situ* density anomalies, revealing the stratification within the water column. **(E,F)** Temperature measurements of the whole water column during transect samplings.

The Pricken time series station at the mouth of the Gullmar Fjord showed highly dynamic changes in both abiotic and biotic factors (Figure 1). The mean salinity over the time series ranged between 20 PSU in spring 2016 and a high of 34 PSU in the fall (Figure 2A). The greatest change in salinity was a 10 PSU increase at the end of September, at which point the salinity was higher than all previous observations and remained elevated until January 2017. Temperature increased from 2.5°C in winter to a summer peak of 20.3°C (Figure 2B). The most rapid changes in temperature occurred in mid-spring (an increase of 5.8°C) and fall (a decrease of 7.2°C).

The summer KB1707 and early fall KB1709 transects included 7 stations (St 1–St 7) stretching from an up-fjord freshwater riverine station to an offshore station 5 km from the mouth of the fjord (Figure 1). The summer transect showed a minimum salinity of 27 PSU at the surface with a halocline between 10 and 17 m and deep-water salinities of 33–35 PSU (Figure 2C). In early fall, there was a freshening of the surface layer to 19–24 PSU with a shallower and more pronounced halocline (Figure 2D) compared to the summer. Summer transect temperatures ranged from 7°C in the deep water to 18°C at the surface and 20°C at St. 1 (Figure 2E). Fall observed lower surface temperatures than summer, and the temperature was uniform down to 45 m (Figure 2F). Three density layers were observed within the water column, with the boundary between the lower and middle layer occurring at 50 m depth during both transects (Figures 2C,D). The depths of the surface-middle layer boundaries, however, differed between the two transects. The summer pycnocline occurred around 18 m at St. 2 and became shallower toward the mouth of the fjord. The pycnocline during fall was at 10 m depth over the length of the fjord with a stronger degree of stratification.

Time series nutrient concentrations exhibited seasonal dynamics (Supplementary Figures 1A,B). The NH_4_^+^, NO_3_^–^ + NO_2_^–^, PO_4_, and SiO_2_ concentrations were highest in winter and sharply decreased into spring with a small increase again in mid-spring 2016. Summer also held minimal nutrient inventories, which increased again into the winter. During KB1707, summer nutrients were depleted in the surface waters of the fjord, with the exception of NH_4_^+^ at St. 5 and 6 (Supplementary Figures 2A,C,E,G). Fall nitrate-nitrite, and phosphate, concentrations had increased at 15 m but were still deplete in the surface waters, while NH_4_^+^ and SiO_2_ concentrations increased at 15 m and the surface (Supplementary Figures 2B,D,F,H).

### Total Bacterial Abundances

Flow cytometry measurements of total bacterial abundances in the time series ranged between 0.9 and 3.7 × 10^6^ cells ml^–1^ (Supplementary Figure 3A). Peaks in abundance were observed in late spring and late summer 2016, as well as spring 2017. Summer abundances were also generally higher than spring, fall, and winter. During the summer transect, bacterial abundances throughout the fjord ranged from 1.1 to 2.0 × 10^6^ cells ml^–1^ (Supplementary Figure 3B). The fall transect had abundances of up to 4.5 × 10^6^ cells ml^–1^ in the surface waters and abundances decreased with depth (Supplementary Figure 3C).

### Total Chl *a* Concentration and Picophytoplankton Abundances

**FIGURE 3:**
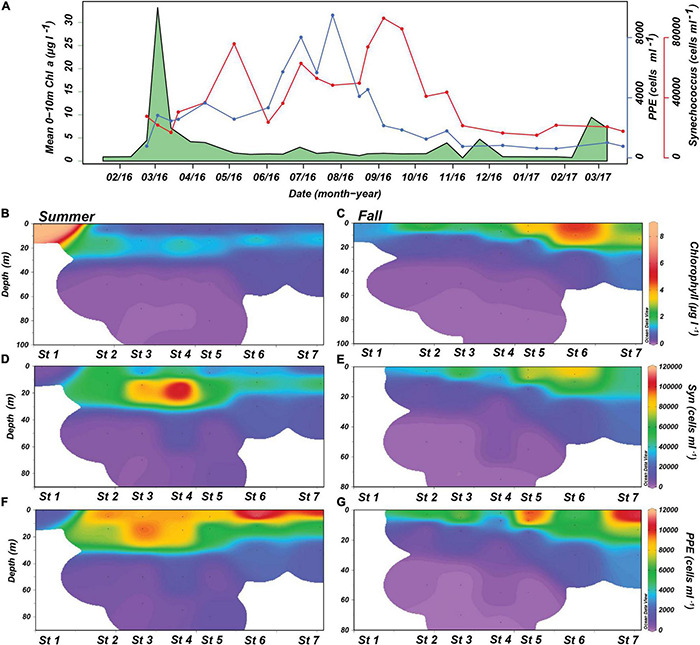
Picophytoplankton and chlorophyll *a* (Chl *a*) concentrations during the time series, summer, and fall transects. **(A)** Mean Chl *a* concentrations from 0 to 10 m, photosynthetic picoeukaryote (PPE) and *Synechococcus* cell abundances over the 2016–2017 time series. **(B,C)** Chl *a* concentrations, **(D,E)** *Synechococcus* cell abundances, and **(F,G)** PPE cell abundances from the KB1707 and KB1709 transects.

Throughout the time series, phytoplankton biomass (indicated by measured Chl *a* concentrations) changed seasonally accompanied by shifts in abundances of picophytoplankton groups (Figure 3A). The Chl *a* concentration was highest in early spring, with a maximum of 33.2 μg l^–1^ during the spring bloom. This concentration decreased into the summer where it remained below 4 μg l^–1^ until mid-fall (October). Through winter, Chl *a* concentration remained <1 μg l^–1^ until February, when the concentration reached 9.5 μg l^–1^. *Synechococcus* had three distinct abundance peaks observed in May, June, and September 2016, with the late summer peak being the largest (up to 9.3 × 10^4^ cells ml^–1^) and longest lasting. This peak was six times higher than the observed minimum in January 2017 at 1.5 × 10^4^ cells ml^–1^ indicating that *Synechococcus* was present at relatively high cell abundances throughout the year. *Synechococcus* abundances were also positively correlated to total bacteria concentration throughout the year (*r*^2^ = 0.59) (Supplementary Figure 3D). The PPE had a smaller abundance peak in April, 3 weeks before the first *Synechococcus* peak, and had a subsequent abundance maximum in June and July at 9.4 × 10^3^ cells ml^–1^. Following these peaks, abundances decreased to 6.2 × 10^2^ cells ml^–1^ in winter.

During the summer transect, Chl *a* was highest at riverine St. 1 (9.2 μg l^–1^) (Figure 3B). Within the fjord, there was a subsurface Chl *a* maximum at 15 m (∼1.5 μg l^–1^) that extended from St. 2 to St. 7. The fall Chl *a* profiles had the highest concentrations accompanying the fresher and more stratified waters at the surface (Figures 2D, 3C). Chl *a* was also higher near the mouth of the fjord in fall, with a maximum of 4.9 μg l^–1^ at St. 6, and a minimum observed at St. 1 (1.08 μg l^–1^). Chl *a* values at 50 m and below were generally <0.1 μg l^–1^ in both transects.

During summer, *Synechococcus* was most abundant at the bottom of the upper mixed layer (15 m), and had a maximum of 1.1 × 10^5^ cells ml^–1^ at St. 4 (Figure 3D). Abundances also decreased toward the mouth of the fjord. Lowest *Synechococcus* abundances (3.2 × 10^3^ cells ml^–1^) were observed at the bottom of the water column and at the riverine station. The highest abundances during fall were observed closer to the surface with a maximum of 8.4 × 10^4^ cells ml^–1^ at St. 6 and were generally lower than summer with a minimum of 1.1 × 10^3^ cells ml^–1^ at 70 m (Figure 3E). However, unlike the summer observations, *Synechococcus* abundances were higher toward the mouth of the fjord in fall (Figures 3D,E).

Summer PPE abundances were greatest at 5 m except for St. 3 (Figure 3F). The highest abundances were observed toward the mouth of the fjord (St. 7), with a maximum of 1.2 × 10^4^ cells ml^–1^ at St. 6. Similar to *Synechococcus*, abundances dropped significantly for measurements below 15 m. The riverine station also had relatively low PPE abundance of 1.6 × 10^3^ cells ml^–1^. During fall, PPE abundances were lower than summer but still had their highest abundances observed around 5 m with the exception of St. 6 (Figure 3G).

### *Synechococcus* Amplicon Sequence Libraries

Of the 7,964,473 bacterial 16S rRNA gene reads, 15% were classified as cyanobacteria and 97.6% of those cyanobacterial sequences were in the order Synechococcales, consisting of 239 unique ASVs (Supplementary Tables 1, 2). On average, there were 10,030 reads per sample classified as Synechococcales (Max: 110,119; Min: 22). Throughout the time series, Synechococcales contributed most of the gene reads in early summer, with as high as 46% of the total reads. In the transects, the highest relative contributions to the total reads (77 and 75%, respectively) were observed above 20 m in the water column.

### Phylogenetic Placement of Amplicon Sequence Variants

**FIGURE 4:**
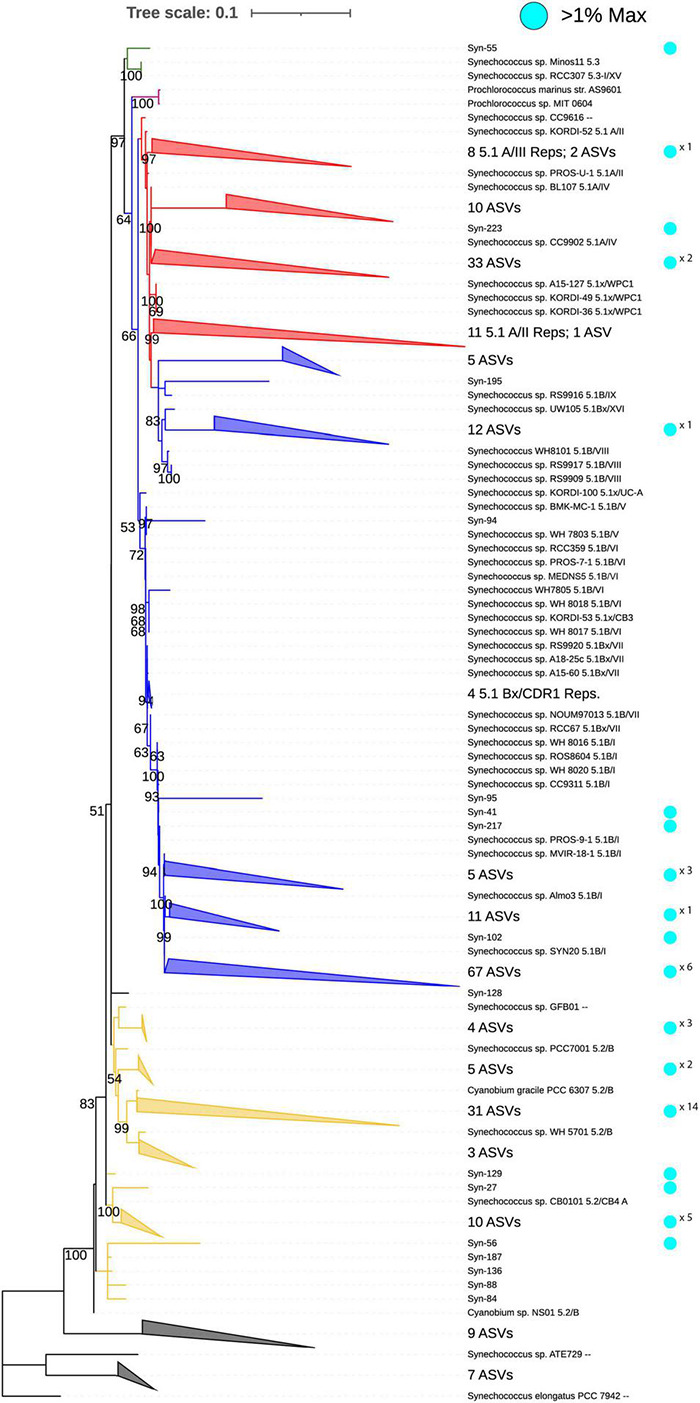
Phylogenetic tree showing 231 environmental Synechococcales (Syn) 16S rRNA gene ASVs (V3–V4 subunit) placed within the full 16S reference phylogeny of 72 *Synechococcus* strains representing subclusters 5.1, 5.2, 5.3 and freshwater isolates as well as *Prochlorococcus* (violet). Branch colors indicate the subcluster classification for 5.2 (yellow), 5.3 (green), 5.1A (red), 5.1B (blue). Black branches indicate freshwater strains. Cyan circles indicate ASVs that composed >1% of the data in an individual sample library. For collapsed branches, cyan circles with × *n* indicate the number of ASVs within that meet the criteria. Bootstrap values (1,000 replicates) are shown for a range of 0–100, only showing values >50.

The phylogeny of the full-length reference 16S rRNA gene sequences of 72 *Synechococcus* strains showed a separation into several major groups (Figure 4). The group containing the largest number of ASVs was 5.1 subcluster with a separation into the 5.1A and 5.1B clades (Figure 4). The 26 subcluster 5.1A reference sequences were present on a single branch that separated them from 5.1B references, with the exception of an internal branch that contained all 5.1B/VIII, IX, and XVI strains. A total of 46 ASVs from this study aligned with references in the 5.1A branch. In turn, 105 ASVs aligned to 5.1B references, with 67 ASVs most similar to SYN20 (5.1B/I).

A second group contained 60 ASVs aligned to references from subcluster 5.2 (Figure 4). Each 5.2 reference recruited multiple ASVs, with the majority being most similar to PCC 6,307 (5.2/B). Only one ASV aligned with subcluster 5.3 references. *Prochlorococcus* ASVs were not present in our dataset, as the two *Prochlorococcus* sp. reference sequences branched separately from all ASVs. Near the root, 16 ASVs were recruited by outgroup terrestrial and fresh water references of *Synechococcus*.

Overall, only a small portion of the detected ASVs reached elevated relative abundances in the fjord. Accordingly, 46 of 239 ASVs were observed to represent >1% of the Synechococcales within any single sample (Figure 4). The ASVs with the greatest representations were Syn-102, Syn-223, and Syn-27, which represented up to 100, 67, and 53% of the Synechococcales reads in an individual sample, respectively (Supplementary Table 1). These three ASVs belonged to subcluster 5.1B, 5.1A, and 5.2, respectively, and had the highest mean representation across all samples collected. Syn-102 had 100% identity to marine strain SYN20 (Accession number CP047959), Syn-223 had 100% identity to marine strain CC9902 (Accession number CP000097), and Syn-27 had 100% identity to fresh water strain MW73B4 (Accession number AY151250). Many other ASVs in both subcluster 5.1 and 5.2 contributed significantly to the *Synechococcus* community in at least one sample, but on average had very low representation.

### Synechococcus Relative Abundances Within the Gullmar Fjord

**FIGURE 5:**
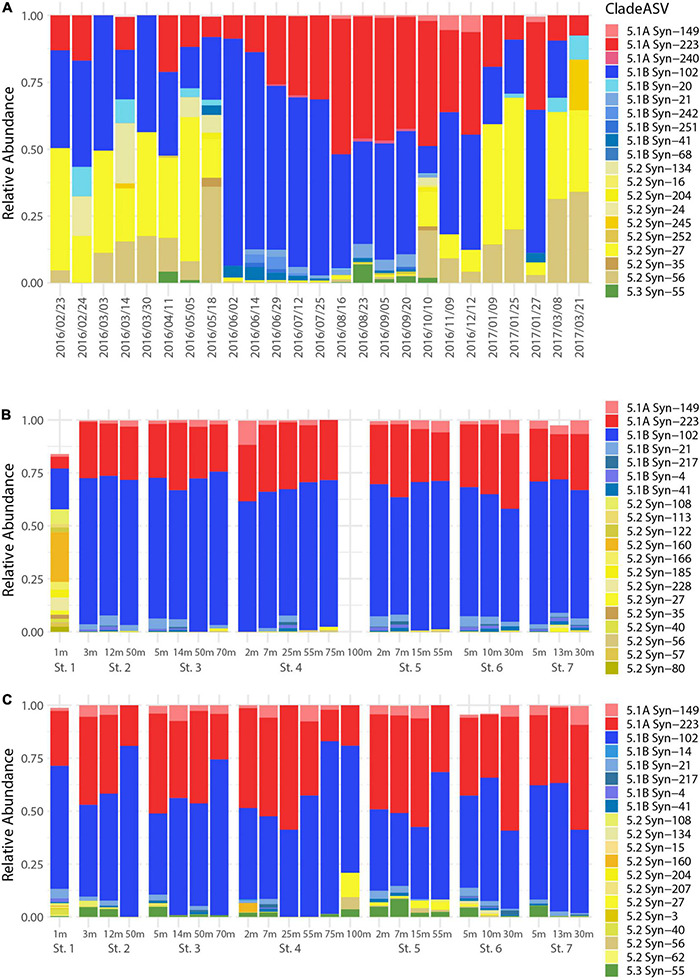
Relative abundances and assigned subclusters depicting the top 20 most abundant Synechococcales ASVs in the **(A)** 2016–2017 time series, **(B)** summer transect (KB1707), and **(C)** fall transect (KB1709). ASVs are assigned to subclusters 5.1A (red), 5.1B (blue), 5.2 (yellow), and 5.3 (green). For **(A)**, each date indicates *n* = 1 libraries. For **(B,C)** at each station/depth, *n* = 2 averaged libraries.

The relative abundances of the 20 most abundant *Synechococcus* ASVs in the time series and transect datasets (Figure 5) were explored among total Synechococcales reads. Four of these ASVs were commonly highly represented (i.e., Syn-27, Syn-56, Syn-102, and Syn-223). In the time series, the changing relative abundances of *Synechococcus* ASVs showed a pattern that corresponded to the changes in *Synechococcus* cell abundances (Supplementary Figure 4A) with two major shifts in relative abundances (Figure 5A). The first shift occurred in spring and the second shift in the fall, which corresponded closely to the start of the second and the end of the third cell abundance peaks in the time series. Before the first shift, Syn-27 and Syn-102 were the dominant ASVs, with Syn-56 and Syn-223 also abundant. Between May and June, Syn-102 rapidly increased in relative abundance while Syn-56 and Syn-27 decreased. Meanwhile, Syn-223 gradually increased through this phase and reached a maximum relative abundance in August. Following the second shift, observed in both ASV assemblage and cell abundances, Syn-56 and Syn-27 increased to dominance again while Syn-223 and Syn-102 decreased; however, Syn-102 was intermittently observed as the dominant ASV also in this period (Figure 5 and Supplementary Figure 4A).

Using the phylogenetic tree placement to determine *Synechococcus* subcluster assignment, it is apparent that the changes in the ASV assemblage corresponded to shifts in dominance between subcluster 5.1 and 5.2 (Figure 5A). The first period showed a dominant and increasing relative abundance of ASVs in subcluster 5.2 that occurred until the shift in spring. The community then changed to dominance of ASVs in subcluster 5.1, with emphasis on 5.1B, while 5.1A gradually increased. Following the second shift, the community dynamics were more variable, with 5.1 and 5.2 subclusters alternating dominance between locations (Figure 5A). The single ASV in subcluster 5.3 only appeared in early spring and late summer/early fall.

Examination of the distribution of *Synechococcus* ASVs during the summer and fall transects uncovered a dominance by two ASVs, Syn-102 and Syn-223, in the fjord, with some differences between summer and fall (Figures 5B,C). Syn-102 made up ∼70% of the sequences throughout the fjord during summer, with steady or slightly higher relative abundance occurring in the deeper depths (50 m and below). Changes with depth were more prominent in the fall transect, with Syn-102 increasing by up to 30% with depth (e.g., St. 4, 25–75 m). Syn-223 had a lower relative abundance than Syn-102 in summer accounting for 25–30% of the sequences, but commonly made up 40–50% of the relative abundance in fall, and decreased in dominance from 50 m and deeper. However, this change in relative abundance was independent of the reduction in cell abundance that occurred below the surface layer (Supplementary Figures 4B,C). The riverine St. 1 contained the greatest ASV diversity observed in this study, with 48 ASVs during KB1707 but only 24 ASVs during KB1709 (Supplementary Figure 5).

In both the summer and fall transects, the fjord was dominated by the 5.1 subcluster which accounted for 80–100% of the sequences while subcluster 5.2 made up <20% (Figures 5B,C). The exception to this was at St. 1 in summer, where 5.2 was the dominant subcluster accounting for 71% of the sequences. The 5.1B subcluster increased in relative abundance at and below 50 m during fall, following the trend of its major ASV contributor Syn-102, but this trend was not observed with consistency during summer. Subcluster 5.3 was only observed during the fall transect but was present at all stations, and represented <10% of the community at any given depth.

**FIGURE 6:**
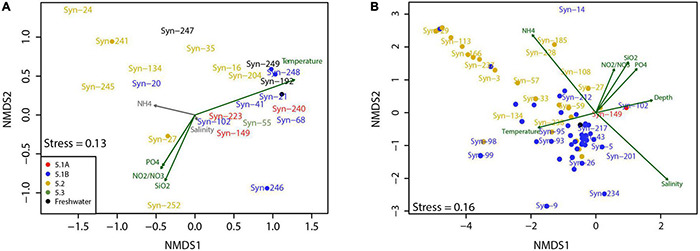
Non-metric multidimensional scaling (NMDS) ordination of ASV relative abundance Bray–Curtis dissimilarities from **(A)** time series and **(B)** transects datasets. Points indicate overlapping ASVs in the ordination space. Environmental parameters with a significant correlation (*P* < 0.05) to ordinations are shown in green.

Ordination of the ASVs by community composition was used to further investigate the environmental associations in our time series and transects datasets (Figure 6A). While there were no distinct groups that formed in the time series separating 5.1 and 5.2 subclusters, the subcluster 5.2 ASVs inhabited a larger area in the ordination space than 5.1. Temperature and NO_3_^–^ + NO_2_^–^ and SiO_2_ concentrations were significantly correlated with the ordination. Subcluster 5.1 ASVs tended toward warmer temperatures while 5.2 was not correlated with environmental conditions.

Ordination of the transect communities revealed that ASVs belonging to subcluster 5.1 and 5.2 also shared ordination space when examining the whole fjord; however, these subclusters were spread differently throughout the space (Figure 6B). Many 5.2 ASVs were associated with lower salinity and higher NH_4_^+^ while few 5.1 ASVs shared this association. Subcluster 5.1 ASVs associated most with warmer temperature and higher salinities. Meanwhile, the two ASVs from 5.1A both associated with cooler temperatures and increasing depth. All environmental variables showed significant correlation to the ordination.

## Discussion

*Synechococcus* in the Gullmar Fjord showed abundance dynamics that varied seasonally and differed from the PPE community within the fjord, suggesting they play a unique function in the fjord’s ecology. With elevated abundance throughout the late spring and summer, culminating in a late summer peak, *Synechococcus* abundance appears to coincide with elevated temperatures within the fjord, as well as elevated bacterial abundance. Accumulation of *Synechococcus* in summer occurred when temperatures rapidly increased between 10 and 15°C, suggesting that warmer waters play an important role in their development. Kuylenstierna et al. (1994) also observed *Synechococcus* abundance over a year long time series in the fjord, revealing a similar occurrence, where the *Synechococcus* increased in summer once temperature reached around 10°C and subsequently decreased once temperatures again reached 10°C in fall. The similarity in the abundance dynamics between *Synechococcus* and total bacteria may be driven by favorable growth conditions to both (Li, 1998). *Synechococcus* may also be taking advantage of nutrient cycling occurring during periods of elevated bacterial abundance (Weinbauer et al., 2011). These observations do not extend to the PPE dynamics in the fjord, which decreased in abundance throughout the summer as *Synechococcus* concentrations increased, and did not coincide with elevated bacteria abundances. As the dominant picophytoplankton present in the late summer, *Synechococcus* may play a greater role in feeding higher trophic levels at this time. Rotifers, copepod nauplii, and cladocerans in the Baltic Sea have all been shown to consume substantial quantities of picocyanobacteria, with consumption increasing with both higher abundance of picocyanobacteria and lower abundances of alternative prey (Motwani and Gorokhova, 2013).

### Divergence in Seasonal Dynamics Within *Synechococcus* Subclusters

The diversity of *Synechococcus* present in the Gullmar fjord encompasses representatives of both the marine 5.1 subcluster as well as those from 5.2 as major players in the system, with the dominant representatives alternating through the annual cycle. From both the time series and the transects, it appears that members of the 5.1 subcluster are primarily dominant in the summer. This abundant summer community is largely composed of co-occurring clade IV (Syn-223) and clade I (Syn-102) ASVs, suggesting these ASVs occupy a similar niche space. The presence of these clades in the fjord is both consistent with their prevalence in high latitude systems (Zwirglmaier et al., 2008) as well as a previous summer time observation in the fjord (Celepli et al., 2017). Between the two, clade I was generally dominant over clade IV and had abundances that could more rapidly change while clade IV abundance more gradually increased and decreased. By August, Syn-223 (clade IV) slightly overcomes Syn-102 (clade I) in abundance. Similar patterns in clades IV and I dynamics have previously been observed in marine systems with clade I starting as the dominant clade during blooms and later shifting to IV (Tai and Palenik, 2009; Nagarkar et al., 2021). This suggests there are either environmental factors that facilitate Syn-102 accumulation and dominance in early summer, which change to favor Syn-223 as the season progresses, or that Syn-102 is more of an opportunist and quickly responded to changes in the environment while Syn-223 more slowly grew to dominance.

While many ASVs were observed within the fjord, over 99% of the *Synechococcus* community at any individual point in time or space was composed of no more than ten ASVs. Moreover, only four ASVs were regularly abundant and alternated as the dominant ASVs throughout the time series, each belonging to a separate clade. In the winter, spring, and autumn months, sustained prevalence of Syn-102 (subcluster 5.1/clade I), Syn-223 (subcluster 5.1/clade IV), Syn-27 (subcluster 5.2), and Syn-56 (subcluster 5.2) suggests niche spaces that allow for coexistence between these ASVs. Together, Syn-27 and Syn-56 made subcluster 5.2 represent over 50% of the sequences in nine of the 16 samplings outside of the summer season. A previous summertime survey of clade diversity ranging from the Bothnian Bay to the Skagerrak suggested that the Gullmar Fjord as well as the Skagerrak and Kattegat host a community dominated by subcluster 5.1, which contrasted from the 5.2 communities of the Baltic Sea (Celepli et al., 2017). Indeed, subcluster 5.1 represented >90% of the community during the summer bloom period in the Gullmar Fjord. However, this dominance subsided in the fall, showing 5.2 clades contributed to community composition similarly to 5.1 for the majority of the year. Given this community shift in the Gullmar Fjord, it is possible that similar community shifts accompany seasonal environmental changes throughout the region.

Seasonal shifts in *Synechococcus* community composition have previously been observed in coastal waters. Mackey et al. (2017) observed clades I and IV dominated in a coastal marsh system in early summer but the community came to include a greater diversity of clades with VI and CB5 in late summer. Tai and Palenik (2009) also observed clades IV and I were dominant over multiple years during the late spring-summer peaks in abundance followed by increased abundances of clades II and III later in the year when total *Synechococcus* abundances were lower. In the Gullmar Fjord, this seasonal shift in community composition appeared to be visible mainly as shifts between members of clades I and IV from subcluster 5.1 and members of 5.2.

The late spring temperature increase in 2016 coincided with a shift to a *Synechococcus* community dominated by subcluster 5.1 ASVs, which sustained dominance throughout the warm period. The community shift that occurred in the following autumn and winter did not coincide with the temperature change as clearly, with subcluster 5.2 becoming prominent again in the month prior to the rapid temperature decrease. Further, while temperatures were well below 10°C in November and December, the subcluster 5.1 clades reemerged as the dominant contributors to the community. This suggests that while temperature is not the only factor influencing shifts in relative abundances, seasonally elevated temperatures appear to help create an environment favoring high abundances and larger representation of 5.1 clades I and IV.

Temperature also drew the greatest response of all environmental variables to shifts in community structure, with relative abundances of some of the less prominent ASVs showing the greatest influence. For both subcluster 5.1 and 5.2, the warm summer temperatures increased the representation of ASVs that were not observed or were very rare during the rest of the year (e.g., Syn-204 and Syn-21). This association with higher temperatures was greater for subcluster 5.1, as only one 5.1 ASV (Syn-20) associated with colder waters.

Coinciding with the drop in *Synechococcus* abundance between September and October, there was a significant 10 PSU increase in salinity that was sustained until the end of January. Based on previous observations in the Baltic Sea region along a salinity gradient (Celepli et al., 2017), this salinity increase would be more likely to shift the community further to a subcluster 5.1 dominated system. However, here we observed that 5.2 was commonly well represented or dominant throughout the winter period at salinities >30 PSU. Salinity therefore does not appear to be limiting the representation of subcluster 5.2 ASVs observed over the time series. It is therefore possible that the observed range of certain 5.2 clades is limited less by salinity tolerance and more by competition from highly competitive clades (i.e., I and IV) during periods with higher salinity.

The composition and abundance of *Synechococcus* likely influences food web dynamics throughout the year in the Gullmar Fjord. Phytoplankton in the Gullmar Fjord are seasonally regulated by top down grazing interactions, with summertime observations of microzooplankton removing a quarter of the standing stock on a daily basis (Tiselius et al., 2016; Arias et al., 2020). Meanwhile, in laboratory settings, selective grazing by nanoflagellates (Zwirglmaier et al., 2009), dinoflagellates, and ciliates (Apple et al., 2011) on *Synechococcus* strains has been shown, with select Synechococcus strains promoting grazer growth and experiencing differences in grazing pressure. Palatability varies widely among *Synechococcus* clades, precluding an evaluation of grazing influence based on clade composition (Zwirglmaier et al., 2009). Strain specificity in viral infection (Suttle and Chan, 1993) also likely influences the nutrient cycling in the fjord. Studies that investigate the specific relationships between grazers, virus infection, and *Synechococcus* composition are necessary to understand the ecosystem pathways influenced by the diverse *Synechococcus* clades observed in the fjord.

### Divergence in Spatial (Along Fjord and With Depth) Distributions of *Synechococcus* Clades

In the year following the time series, the two transects sampled in July and September 2017 both predominantly showed the 5.1 Syn-102 and Syn-223 as the major ASVs of the *Synechococcus* community over the entire length of the fjord and throughout the water column. Given that these were mid-late summer observations with near surface *Synechococcus* concentrations comparable to the peak of the time series abundance, the prevalence of clades I and IV was similar to the time series measurements. This reinforces that these clades compose the abundant summer community. Further, during the fall transect, ASVs from 5.2 made up a greater fraction of the community along the fjord, which was comparable to the autumn return of 5.2 in the time series. The exception was at the riverine station, where the summer sampling revealed the highest diversity observed within our dataset (Fall St. 6 had almost as many ASVs, but with Syn-223 and Syn-102 largely dominant). The riverine *Synechococcus* abundance was very low, and so the environment may allow microdiversity to reveal itself. However, abundance alone could not explain the observed diversity measurements, as measurements below 50 m in the fjord also had low abundances but had a composition more similar to the fjord’s surface community than to the river. The river ASV diversity (St. 1) was also more in line with the fjord in fall, though no cell counts were available here.

During fall, Syn-223 (clade IV) commonly had a maximum relative abundance around 30 m while Syn-102 (clade I) was proportionally dominant below 50 m depth. This trend between the clades was not as clear in the summer, however, a similar pattern has been previously observed at multiple sites off the coast of southern California attributed to seasonal stratification (Nagarkar et al., 2021). We observed that there was a greater degree of stratification below the shallow surface-mixing layer in fall compared to summer, but the deep-water mass showed similar stratification between the two seasons, only deeper by 5 m in September. These results suggest that either Syn-102 can seasonally grow better than Syn-223 deeper in the water column, at depths only sustaining very low cell concentrations, or that there are physiological differences between clades that can result in the propensity of cells in one clade to sink more than another. De Martini et al. (2018) previously showed that *Synechococcus* sinking flux rates in the open ocean are not similar across clades, with members of clade III having higher cell flux rates compared to clade II relative to their standing stocks. If *Synechococcus* assemblages in Gullmar Fjord exhibit differences in sinking flux characteristics, this will also influence nutrient cycling throughout the fjord.

Despite differences in cell abundances observed in the upper 20 m along the transects, with maxima located centrally and toward the mouth of the fjord in summer and fall, respectively, these abundance peaks did not alter the *Synechococcus* community composition. Similarly, there were no major differences in composition moving along the fjord. This is likely because the greatest environmental gradients occurred with depth rather than along the fjord. With residence times of surface waters that are 16–26 days (Arneborg, 2004) it is also likely that the development of spatially dynamic communities within the surface is minimized. While there were no differences between subcluster 5.1 and 5.2 in association with environmental parameters associated with depth (temperature, NO_2_^–^ + NO_3_^–^, PO_4_, and SiO_2_), both subclusters contained ASVs that tended toward shallower/deeper depths. This suggests that the gradient creates niche space, increasing the diversity of cohabiting ASVs.

### Phylogenetic Placement Using the 16S rRNA Gene V3–V4 Region

The 16S rRNA gene V3–V4 region primers used in this study are widely used to explore the total microbial community composition and offered an opportunity to view *Synechococcus* subcluster dynamics in the context of the total bacterial community. These primers have been used frequently to identify the proportion of *Synechococcus* to total bacterial sequences in the Baltic Sea, Kattegat, and Skagerrak (Bertos-Fortis et al., 2016; Hu et al., 2016; Celepli et al., 2017). However, other genes offer higher resolution for discriminating *Synechococcus* clade diversity (Ahlgren and Rocap, 2012; Huber et al., 2019). Still, several previous studies have used the V3–V4 region to determine *Synechococcus* clade assemblage within their studies’ geographic regions, although they have not commented on the general suitability of the gene region for clade identification (Shilova et al., 2017; Li et al., 2019). Indeed, because of the short sequence length of V3–V4 region and the relatively low information content, this region alone is not suitable for reconstructing solid phylogenies to resolve clade level assignments for marine *Synechococcus*. However, by building a phylogeny based on full length 16S rRNA gene reference sequences and then adding the V3–V4 region ASVs by alignment it was possible for us to construct a useful phylogeny. We found that with this method it was possible to match many ASVs in the dataset to clades, with some ASVs having 100% sequence identity to reference sequences; however, some ASVs were not similar enough to be recruited by reference sequences in our dataset. Given the widespread use of 16S rRNA gene V3–V4 region amplicons for studies of ocean and coastal microbial communities, the region is useful for studying datasets of opportunity, or meta-analysis studies, but it is limited in utility for phylogenetic analyses at high resolution.

### Conclusion

*Synechococcus* are an abundant group of picophytoplankton in the Gullmar Fjord that have growth dynamics uncoupled from the picoeukaryotes in the fjord. The *Synechococcus* community hosts a large diversity, yet a handful of ASVs were particularly important for structuring the community over an annual cycle and across the length of the fjord. These ASVs shifted from a mixed 5.2/5.1 in spring to a 5.1 dominant assemblage during the summer months, suggesting that the change was related to the higher temperatures that accompanied the community shift. Unexpectedly, the community shift was associated with seasonal changes and not salinity. Thus, further investigation into the factors that influence *Synechococcus* clade composition in the region and other estuarine systems is warranted. The prominence of the subcluster 5.2 clades I and IV over the length of the transects suggests the community diversity is largely uniform throughout the fjord, however, transect observations in differing parts of the year are needed to confirm this observation. The prominence of these clades also provided examples of consistency with previous observations in high latitude systems (Zwirglmaier et al., 2007, 2008). Nevertheless, across a full year this prominence shifts and a variety of clades contribute to the biodiversity and productivity of this fjord ecosystem.

## Data Availability Statement

The DNA sequence data presented in the study are deposited in the NCBI SRA repository, BioProject PRJNA784784; https://www.ncbi.nlm.nih.gov/bioproject/PRJNA784784. Flow cytometry data are deposited in the FlowRepository under the ID FR-FCM-Z5ZY; http://flowrepository.org/id/FR-FCM-Z5ZY.

## Author Contributions

BP, CB, CMGO, JP, and HF conceived the study. BP, CB, CMGO, and CP-M conducted the field work and together with HF performed lab work. DL conducted bioinformatics processing of the amplicon dataset. DD conducted bioinformatic analysis of the dataset. CPL processed the data, interpreted results, and drafted the manuscript. CL provided expert advice and interpretation. All authors contributed to the writing of the final version of the manuscript.

## Conflict of Interest

The authors declare that the research was conducted in the absence of any commercial or financial relationships that could be construed as a potential conflict of interest.

## Publisher’s Note

All claims expressed in this article are solely those of the authors and do not necessarily represent those of their affiliated organizations, or those of the publisher, the editors and the reviewers. Any product that may be evaluated in this article, or claim that may be made by its manufacturer, is not guaranteed or endorsed by the publisher.

## Footnotes

1
https://sharkweb.smhi.se/

2
https://sourceforge.net/projects/fcsalyzer/

3
https://qiime2.org

4
http://application.sb-roscoff.fr/cyanorak/

5
http://hmmer.org

6
http://itol.embl.de
