# Supplementary material for: Seasonal and Spatial Variations in Synechococcus Abundance and Diversity Throughout the Gullmar Fjord, Swedish Skagerrak
Source: Front Microbiol. 2022 May 9;13:828459. doi: 10.3389/fmicb.2022.828459 (PMC9125215; doi:10.3389/fmicb.2022.828459)
Supplement: Supplementary file 3 [file Data_Sheet_1.DOCX]

**Supplementary Material**


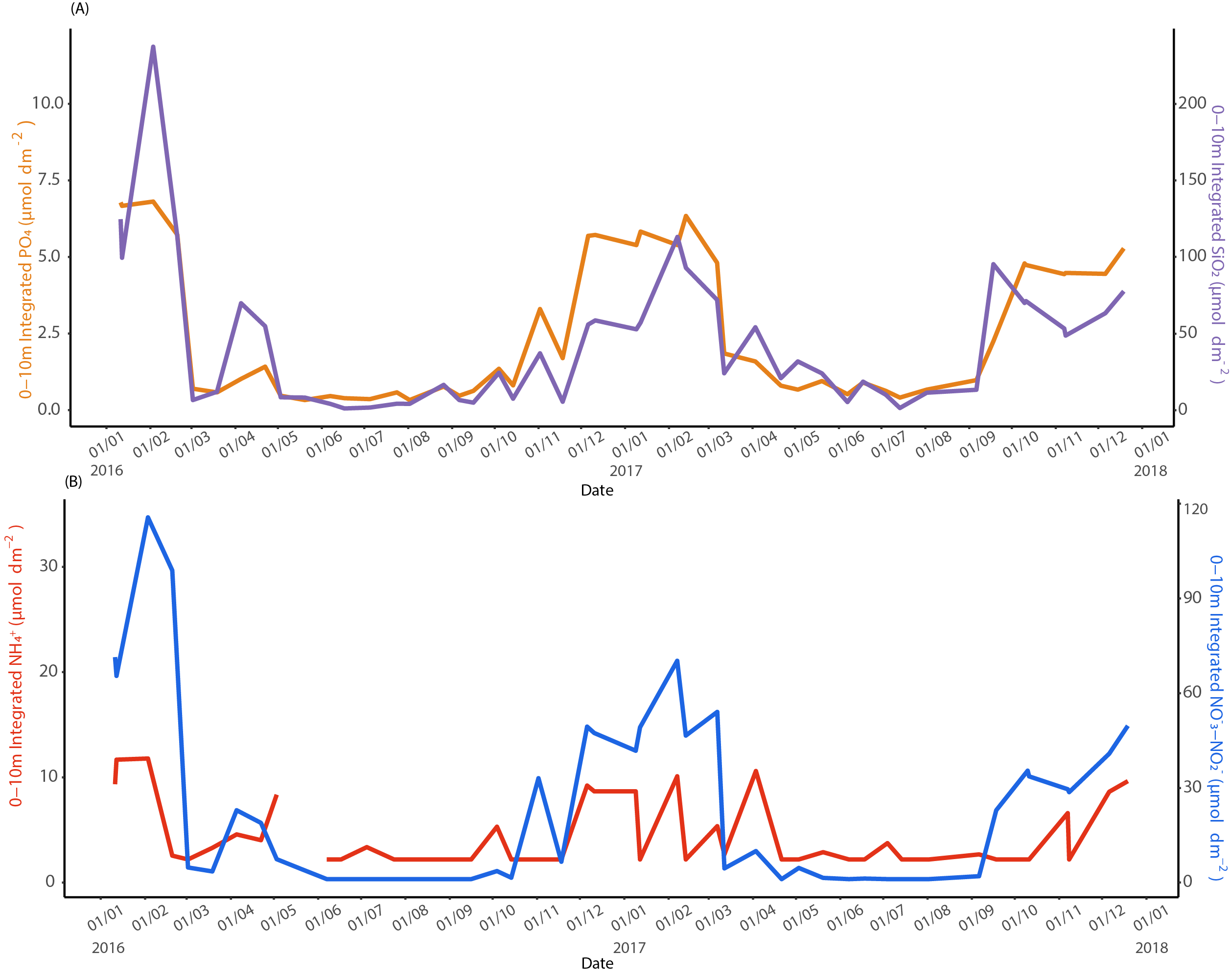


**Supplemental Figure S1**. Depth integrated (0-10 m) nutrient inventories during the 2016-2017 time series. (A) PO_4_ and SiO_2_ (B) NH_4_^+^ and NO^-^_3_-NO^-^_2_.


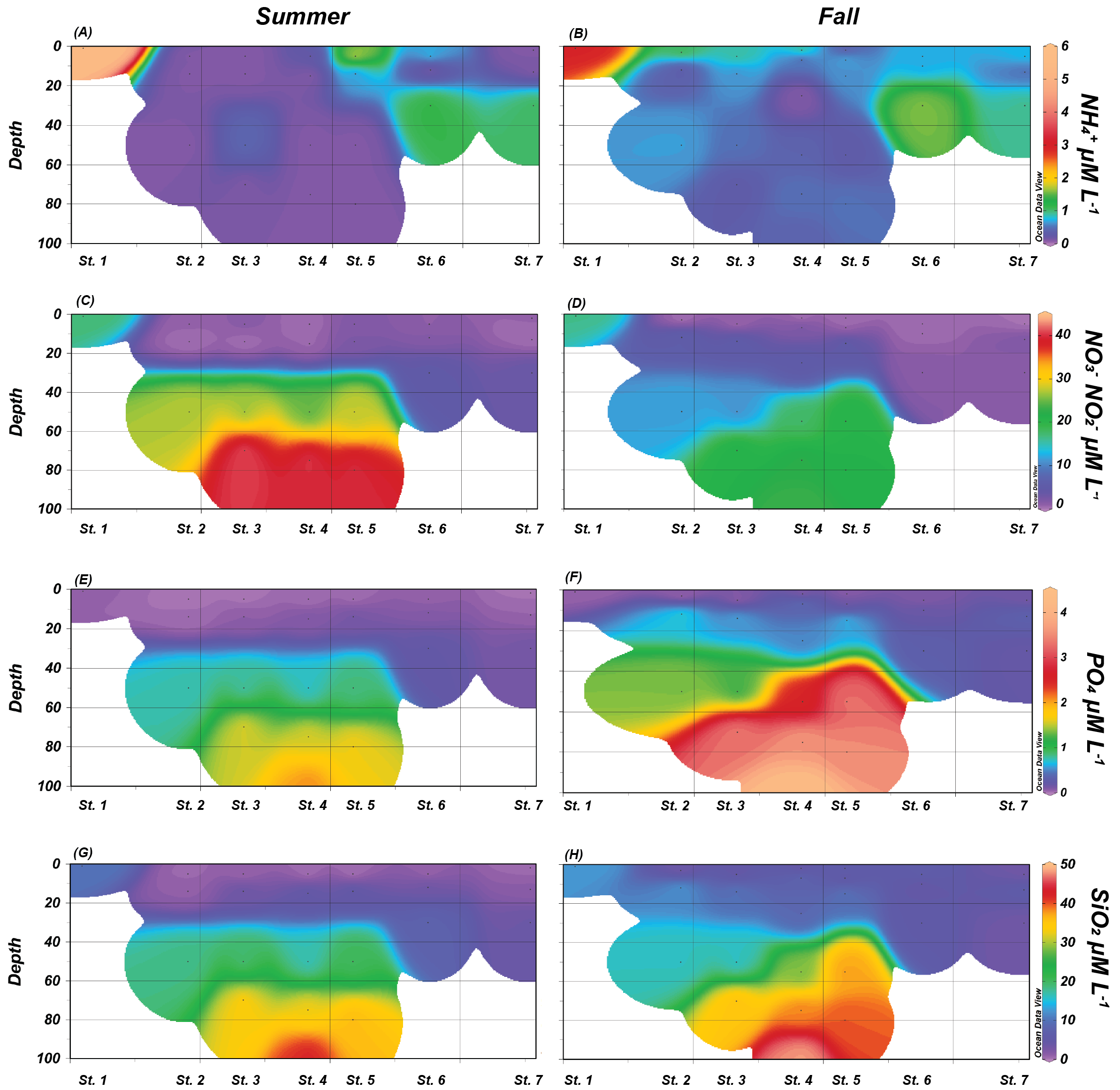


**Supplemental Figure S2**. Nutrient concentrations during the summer and fall transects respectively, (A-B) NH_4_^+^, (C-D) NO_3_^-^-NO_2_^-^, (E-F), PO_4_, and (G-H) SiO_2_ profiles of the water column.


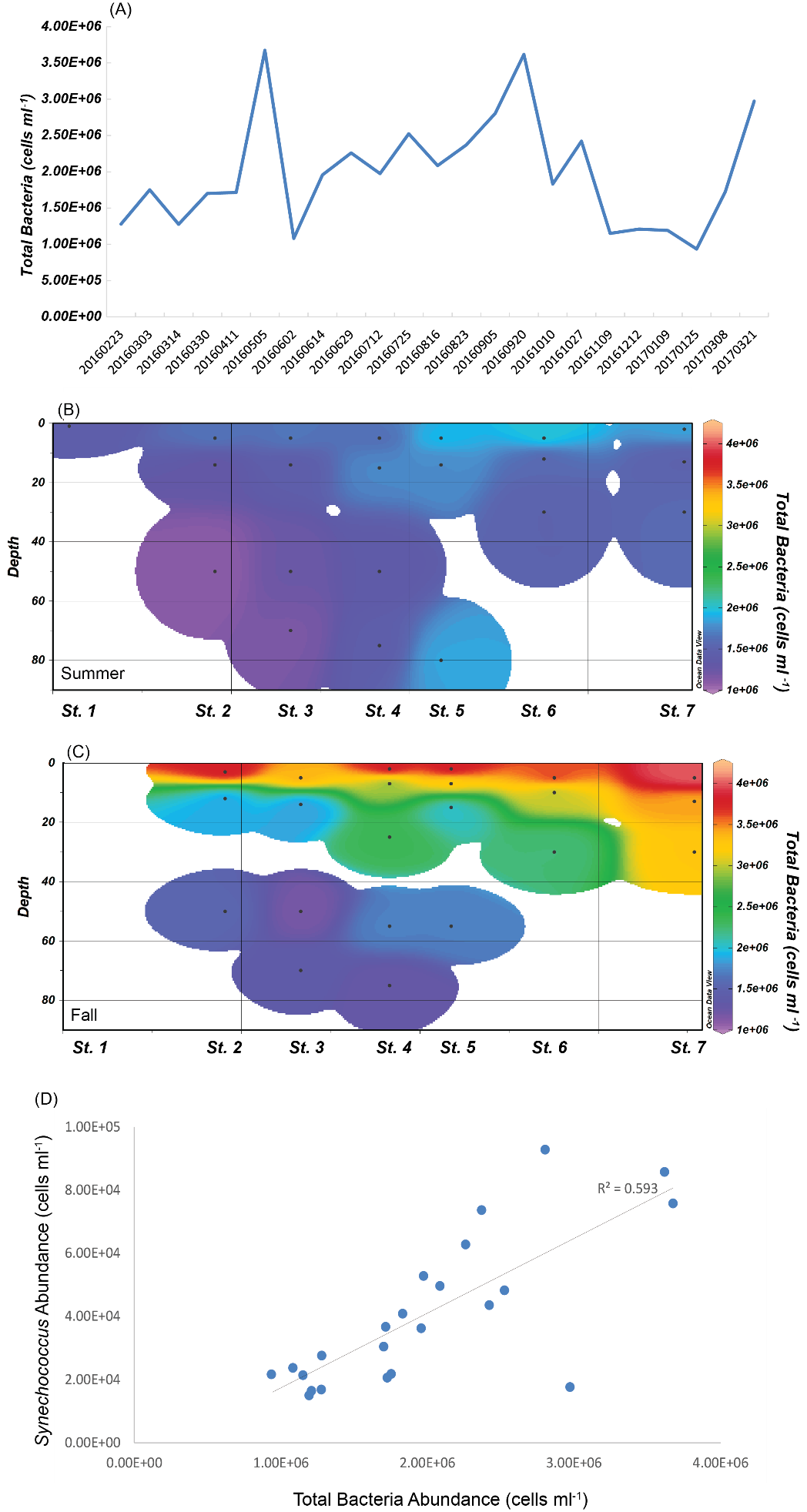


**Supplemental Figure S3**. Total bacterial abundances (A) throughout the 2016-2017 time series and in the (B) KB1707 and (C) KB1709 transects. (D) Correlation between total bacteria and *Synechococcus* abundances throughout the time series.


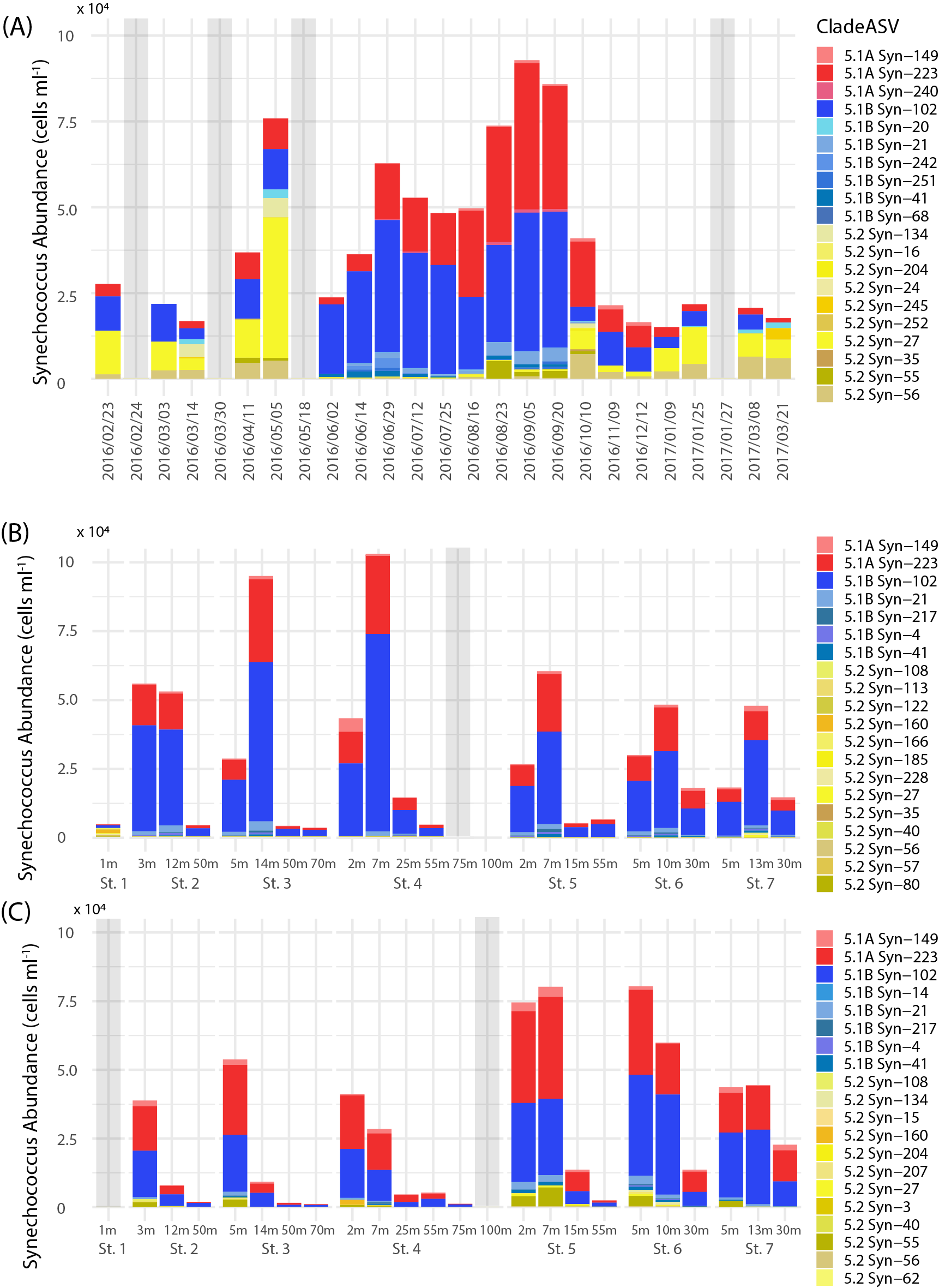


**Supplemental Figure S4**. Abundances and assigned subclusters depicting the top 20 most abundant Synechocococcales ASVs in the (A) 2016-2017 time series, (B) summer transect (KB1707), and (C) fall transect (KB1709). Abundances were estimated by normalizing flow cytometry cell counts to the relative abundances of the ASVs. ASVs are assigned to subclusters 5.1A (red), 5.1B (blue), and 5.2 (yellow). For (A), each date indicates n = 1 libraries. For (B) and (C) at each station/depth, n = 2 averaged libraries. Gray bars indicate dates where no flow cytometry data was collected.


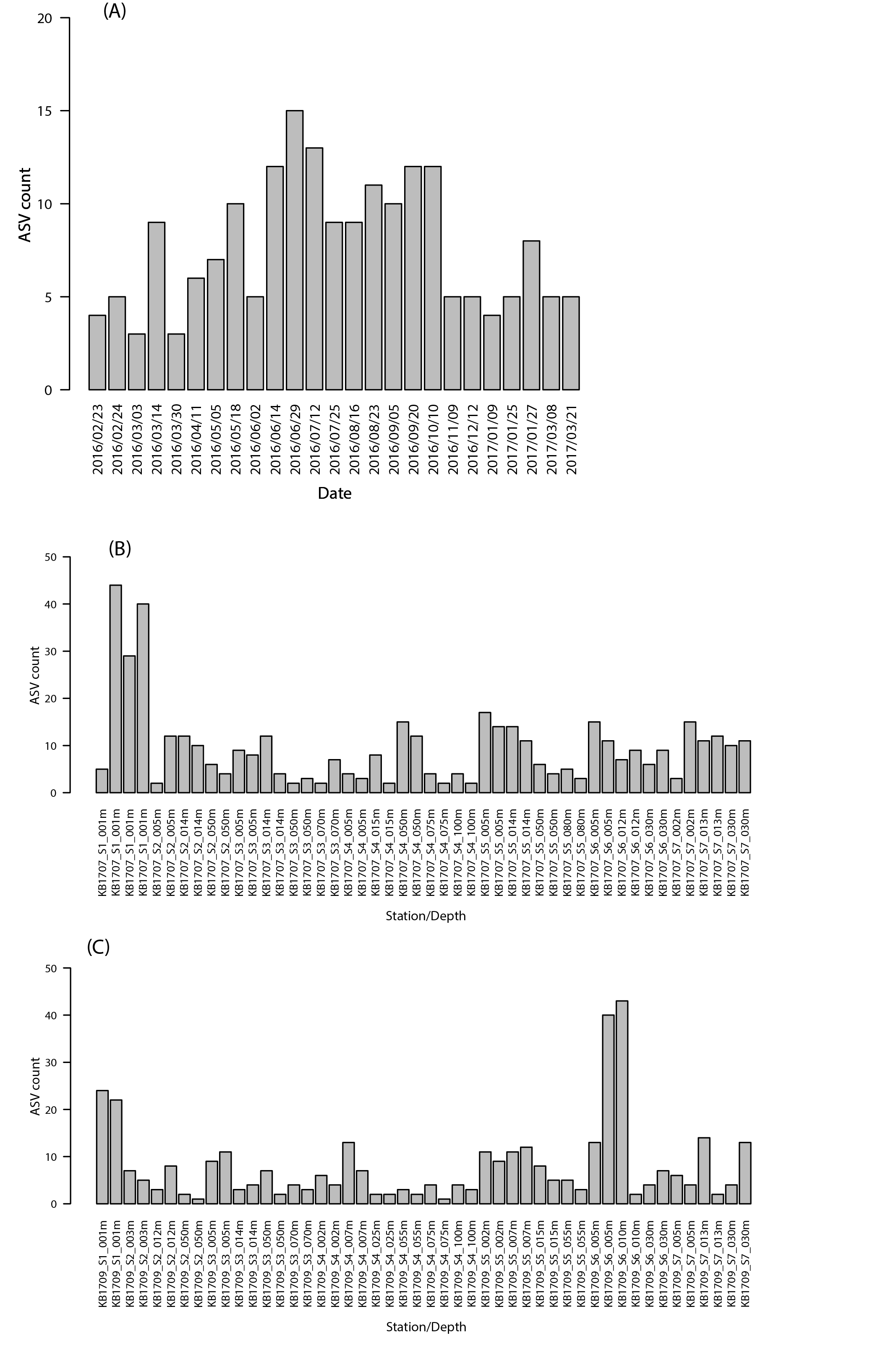


**Supplemental Figure S5**. Number of Synechococcales ASVs identified from each gene library sequenced for this study. For the time series (A) only one sample was collected per station. For the transects (B) KB1707 summer and (C) KB1709 fall duplicate samples were collected for each station/depth, producing duplicate gene libraries.
